# Vision screening of school-aged children in Wuqia County: a cross-sectional study in western China

**DOI:** 10.3389/fmed.2025.1726504

**Published:** 2026-01-08

**Authors:** Yunhui Wu, Xiaoju Hou, Wanchao Zhang, Yishake Kaiseer, Tuerhongbaike Tusunguli, Kelimu Buermahan, Tuxunjiang Xiabire, Jing Ma, Zhibang Hu

**Affiliations:** 1 Department of Eye and ENT, The People’s Hospital of Wuqia, Kezhou, China; 2 Department of Radiology, The People’s Hospital of Wuqia, Kezhou, China; 3 Department of Otorhinolaryngology, Changzhou Third People’s Hospital, Changzhou Medical Center, Nanjing medical University, Changzhou, China

**Keywords:** vision screening, prevalence, reduced visual acuity, school-aged children, western China

## Abstract

**Objective:**

To investigate the prevalence of reduced visual acuity (VA) among school-aged children in Wuqia County, western China.

**Methods:**

In this cross-sectional study, 9,094 primary and junior middle school students in Wuqia County underwent vision screening in November 2024. Uncorrected visual acuity (UCVA) was measured using a LogMAR tumbling E chart, with reduced VA was defined as UCVA < 20/30 in either eye. We also analyzed associations between reduced VA and factors such as gender, grade level, and residential area (urban vs. rural).

**Results:**

The overall prevalence of reduced VA among school-aged children in Wuqia County was 21.52% (1,957/9,094). The prevalence of reduced VA was significantly higher in female children (26.62%, 1,151/4,324) than in males (16.90%, 806/4,770) (*P* < 0.01). The occurrence of reduced VA increased significantly with higher grade levels (*P* < 0.01), from 8.10% in grade 1–31.03% in grade 9. Furthermore, from grade 3 onwards, the prevalence of reduced VA was consistently higher in females compared to males (*P* < 0.05). Additionally, the prevalence of reduced VA was significantly higher in urban primary schools (21.33%, 635/2,977) than in rural primary schools (15.32%, 564/3,682) (*P* < 0.01). Multivariate analysis identified higher grade level, female gender, and urban school location as independent factors associated with an increased risk of reduced VA (all *P* < 0.001).

**Conclusion:**

This study revealed a significant burden of reduced VA among school-aged children in Wuqia County. The findings provide valuable insights for developing targeted interventions and public health policies aimed at improving eye care services in the high-altitude regions of western China.

## 1 Introduction

Vision impairment (VI) constitutes a critical global health issue, imposing a substantial health and economic burden on societies worldwide (1–3). The World Health Organization (WHO) defines VI as a presenting visual acuity (VA) worse than 6/12 in the better seeing eye (4). An estimated 553 million people worldwide were affected by VI in 2020 (5), nearly double the number reported in 2010 (6). Encouragingly, more than 90% of VI cases are attributable to preventable or treatable causes (4). VI in school-age children is particularly concerning, as it can hinder learning, communication, and overall quality of life, with effects that are often lifelong (7, 8). Refractive error, particularly myopia, is the leading cause of VI in children and adolescents and has increased significantly in recent years (9, 10). Consequently, pediatric vision screening programs have been widely implemented globally, including in China, to facilitate early detection and intervention, thereby improving the quality of life for affected children (11–14). Such screenings are crucial not only for preventing VI but also for promoting comprehensive child development (15). Given the high prevalence of uncorrected refractive errors and limited feasibility of cycloplegic refraction in routine school screenings, reduced VA has been widely used as a practical and validated proxy for myopia-related VI in population-based studies (14, 16). According to the American Academy of Ophthalmology, the threshold to pass vision screening aged 60 months and older is the 20/30 line (17). Therefore, reduced VA in this study was defined as UCVA < 20/30 in either eye. The prevalence of reduced VA and VI is influenced by various factors, including economics, geography, and lifestyle (18). For instance, 15.4% of students aged 10 years and older in Mexico exhibited VA of 20/30 or worse in one or both eyes. In Peru, a study found that 6.5% of children presented with VA worse than 20/70 in at least one eye. Among the U.S. population aged 40 years, an estimated 1.98% experience low vision. Meanwhile, in Barbados, the prevalence of VI in the general population was reported at 5.9%. However, no studies have reported vision screening outcomes for school-aged children in Wuqia County, a high-altitude region of western China with limited healthcare infrastructure. Therefore, this study aims to investigate the prevalence of reduced VA to support the early diagnosis and treatment of vision impairment in children in this region.

## 2 Materials and methods

### 2.1 Study population

A total of 9,359 primary and junior middle school students (grades 1–9; aged 6–17 years) were enrolled for the vision screening in Wuqia County in November 2024. 265 children were excluded due to organic eye diseases or other reasons, such as being on leave during the examination period or having transferred to another school. Consequently, 9,094 children underwent vision screening in schools by the trained screening examiners, achieving an overall effectiveness rate of 97.17%.

### 2.2 Vision screening

The vision screening examiners were ophthalmic practitioners, technicians and nurses. And all of them received uniformly trained and qualified. All operations were carried out in accordance with the Technical Specification for Student Health Examination of China (GB/T 26343-2010). UCVA was measured using a LogMAR chart with tumbling E optotypes (Yuanyan Medical) at 5-meter distance. Both eyes were examined separately, starting with the right eye, and then the left eye. Reduced VA was defined as UCVA < 20/30 in either eye. The vision screening examiners subsequently uploaded all the pertinent data to the Comprehensive Myopia Prevention and Control Management System for students in the Xinjiang Uyghur Autonomous Region, in order to establish electronic health records.

### 2.3 Ethical approval

This study was conducted according to the Declaration of Helsinki and approved by the Ethics committee of the People’s Hospital of Wuqia (2024036). This research was affiliated with the National Free Health Check-up Program and the Health Examination Program for Primary and Secondary School Students in the Xinjiang Uyghur Autonomous Region. Prior to the study, informed consent was secured from the parents or guardians of each participating child.

### 2.4 Statistical analysis

All data were analyzed using the IBM SPSS Statistics version27. Quantitative data with a normal distribution were presented as mean ± standard deviation, while descriptive statistics are reported as counts and percentages for categorical variables. The prevalence of reduced VA was compared using the chi-squared test. Multivariate logistic regression analysis was used to evaluate the independent association of the each risk factors (grade, gender, and school location) with reduced VA, and the goodness-of-fit of the logistic regression model was assessed using the Hosmer-Lemeshow test. For the purpose of this study, school location was categorized as urban or rural based on official administrative classifications. Urban schools were defined as those located within the county seat (Wuqia Town). Rural schools were defined as those situated in the surrounding townships and villages of Wuqia County. All statistical tests were two-sided, and *P* < 0.05 was considered statistically significant.

## 3 Results

### 3.1 Characteristics of the study population

The mean age of the participants was 10.87 ± 2.45 years. Among them, 52.45% (*N* = 4,770) were males, and 47.55% (*N* = 4,324) were females. There were no significant age differences between male and female children in any grades. The detailed characteristics of the participants are presented in Table 1. The overall prevalence of reduced VA among all the participants was 21.52% (1,957/9,094). The mean prevalence among males was 16.90% (806/4,770), significantly lower than among females, who had a prevalence of 26.62% (1,151/4,324) (χ^2^ = 126.92, *P* < 0.01).

**TABLE 1:** Demographic details of the participants.

Characteristics		Grade 1	Grade 2	Grade 3	Grade 4	Grade 5	Grade 6	Grade 7	Grade 8	Grade 9	Total
*N*		790	1,076	1,383	1,128	1,166	1,116	848	804	783	9,094
Age, years		6.99 ± 0.26	8.03 ± 0.29	9.19 ± 0.48	10.13 ± 0.51	11.08 ± 0.53	12.09 ± 0.55	13.12 ± 0.52	14.08 ± 0.57	14.97 ± 0.57	10.87 ± 2.45
Gender	Male, n(%)	419(53.04%)	598(55.58%)	723(52.28%)	581(51.51%)	600(51.46%)	588(52.69%)	471(55.54%)	417(51.87%)	373(47.64%)	4,770(52.45%)
	Female, n(%)	371(46.96%)	478(44.42%)	660(47.72%)	547(48.49%)	566(48.54%)	528(47.31%)	377(44.46%)	387(48.13%)	410(52.36%)	4,324(47.55%)
School location	Urban, n(%)	408(51.65%)	498(46.28%)	563(40.71%)	478(42.38%)	545(46.74%)	485(43.46%)	–	–	–	2,977(44.71%)
	Rural, n(%)	382(48.35%)	578(53.72%)	820(59.29%)	650(57.62%)	621(53.26%)	631(56.54%)	–	–	–	3,682(55.29%)

### 3.2 The prevalence of reduced VA across grades

**FIGURE 1:**
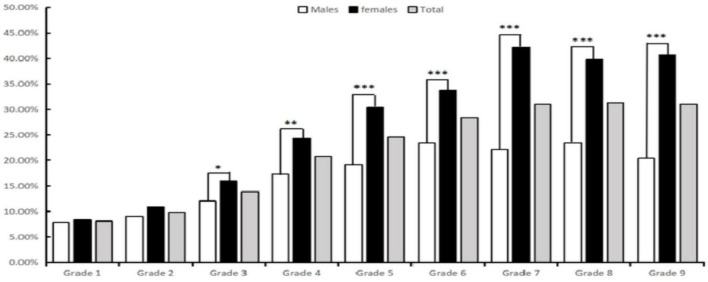
The prevalence of reduced VA in each grade. *P < 0.05; **P < 0.01; ***P < 0.001.

As shown in Table 2 and Figure 1, the prevalence of reduced VA was increased with grade level in a non-linear manner, from 8.10% in grade 1–31.03% in grade 9. This difference was statistically significant (χ^2^ = 387.69, *P* < 0.001). Similar trends were observed in both males (χ^2^ = 895.51, *P* < 0.001) and females (χ^2^ = 304.81, *P* < 0.001). Additionally, from grade 3 to grade 9, the prevalence of reduced VA were consistently higher among females compared to males (χ^2^ = 4.34, *P* = 0.037; χ^2^ = 8.23, *P* = 0.004; χ^2^ = 19.77, *P* < 0.001; χ^2^ = 14.38, *P* < 0.001; χ^2^ = 39.52, *P* < 0.001; χ^2^ = 24.76, *P* < 0.001; χ^2^ = 37.81, *P* < 0.001; respectively).

**TABLE 2:** The prevalence of reduced VA across grades.

Gender	Grade 1	Grade 2	Grade 3	Grade 4	Grade 5	Grade 6	Grade 7	Grade 8	Grade 9	Chi-squared (χ ^2^)	*P-*value
Male, n(%)	33(7.88%)	54(9.03%)	87(12.03%)	101(17.38%)	115(19.17%)	138(23.47%)	104(22.08%)	98(23.50%)	76(20.38%)	895.51	< 0.001
Female, n(%)	31(8.36%)	52(10.88%)	105(15.91%)	133(24.31%)	172(30.39%)	178(33.71%)	159(42.18%)	154(39.79%)	167(40.73%)	304.81	< 0.001
Total, n(%)	64(8.10%)	106(9.85%)	192(13.88%)	234(20.74%)	287(24.61%)	316(28.32%)	263(31.01%)	252(31.34%)	243(31.03%)	387.69	< 0.001

### 3.3 Reduced VA in different regions

**FIGURE 2:**
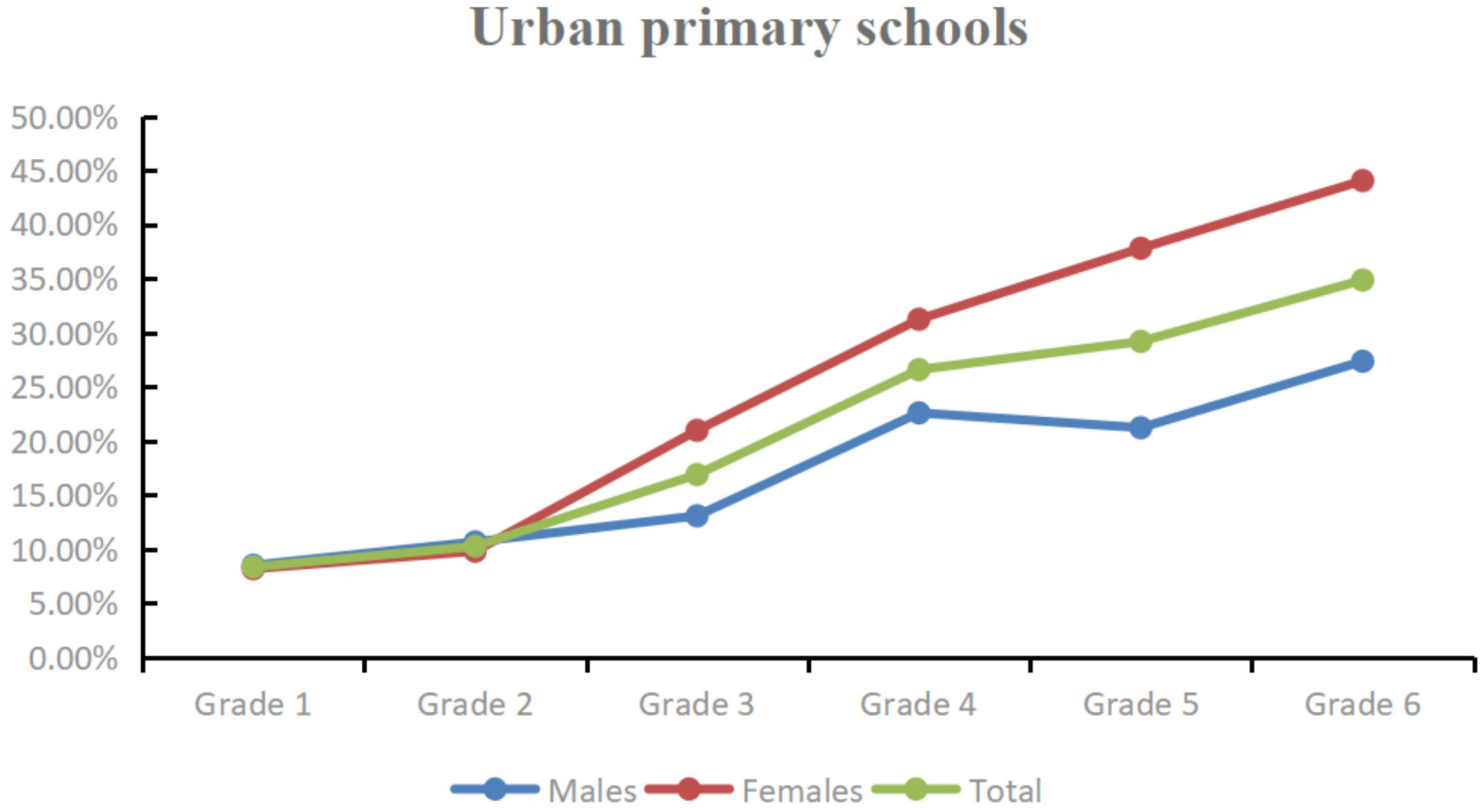
The prevalence of of reduced VA in urban primary schools.

**FIGURE 3:**
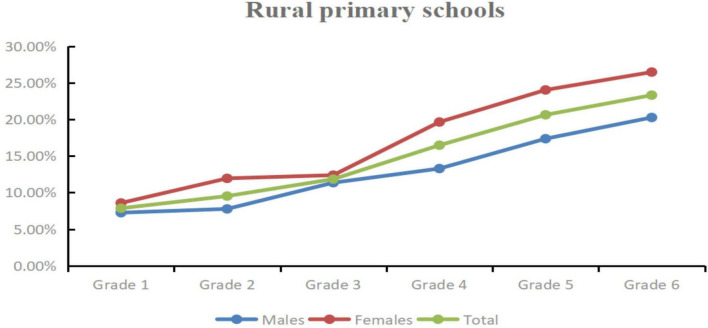
The prevalence of of reduced VA in rural primary schools.

Given that both junior high schools were located in Wuqia Town. Approximately 80% of junior high school students resided on campus, sharing similar living conditions such as boarding facilities, study environments, and daily routines. This homogeneity limited the analysis of potential urban–rural disparities at this educational level. Consequently, our analysis focused solely on the disparities in reduced VA prevalence between urban and rural primary schools. The average prevalence of reduced VA among primary schools was 18.01% (1,199/6,659). Notably, the prevalence of reduced VA in urban primary schools was 21.33% (635/2,977), significantly higher than that in rural primary schools, which was 15.32% (564/3,682) (χ^2^ = 40.31, *P* < 0.001). Furthermore, in both urban and rural primary schools, there was a graded increase in the prevalance of reduced VA among both males and females (Urban males: χ^2^ = 49.66, *P* < 0.001; Urban females: χ^2^ = 128.08, *P* < 0.001; Rural males: χ^2^ = 35.31, *P* < 0.001; Rural females: χ^2^ = 48.32, *P* < 0.001) (Table 3 and Figures 2, 3).

**TABLE 3:** The prevalence of of reduced VA in different regions.

School location		Grade 1	Grade 2	Grade 3	Grade 4	Grade 5	Grade 6	Chi-squared (χ^2^)	*P-*value
Urban, n(%)		34(8.33%)	51(10.24%)	95(16.87%)	127(26.57%)	159(29.17%)	169(34.85%)	164.82	< 0.001
Rural, n(%)		30(7.85%)	55(9.52%)	97(11.83%)	107(16.46%)	128(20.61%)	147(23.30%)	84.14	< 0.001
Urban	Male, n(%)	18(8.49%)	28(10.65%)	38(13.06%)	58(22.57%)	60(21.20%)	73(27.34%)	49.66	< 0.001
	Female, n(%)	16(8.16%)	23(9.79%)	57(20.96%)	69(31.22%)	99(37.79%)	96(44.04%)	128.08	< 0.001
Rural	Male, n(%)	15(7.25%)	26(7.76%)	49(11.34%)	43(13.27%)	55(17.35%)	65(20.25%)	35.31	< 0.001
	Female, n(%)	15(8.57%)	29(11.93%)	48(12.37%)	64(19.63%)	73(24.01%)	82(26.45%)	48.32	< 0.001

### 3.4 Multivariate logistic regression analyses for reduced VA

Multivariate logistic regression analysis was conducted to evaluate potential risk factors for reduced VA while controlling for confounding variables. The *P*-value of the Hosmer-Lemeshow test was 0.099 (χ^2^ = 13.391), indicated that the logistic regression model provides an adequate fit to the observed data. Table 4 showed higher grade, female and residence in urban were considered as significant independent risk factors for reduced VA (*P* < 0.001). Among these findings, the risk of reduced VA in grade 3 was nearly twice compared to that in grade 1, with an odds ratio (OR) of 1.93 (95% confidence interval (CI): 1.44–2.62, *P* < 0.001). By grade 6, the risk had escalated to 4.74 times compared with grade 1 (OR 4.74, 95%CI: 3.58–6.38, *P* < 0.001). Furthermore, female children faced a 1.54 times higher risk of reduced VA compared to male children (OR 1.54, 95% CI: 1.35–1.75, *P* < 0.001), while children from urban areas had a 1.57 times increased risk compared to those from rural areas (OR 1.57, 95% CI: 1.38–1.79, *P* < 0.001).

**TABLE 4:** Multivariate logistic regression analysis for the factors associated with reduced VA.

Characteristic	OR	95% CI	*P*-value
**Grade**			
1	Reference		
2	1.28	0.93, 1.79	0.13
3	1.93	1.44, 2.62	< 0.001
4	3.12	2.34, 4.22	< 0.001
5	3.83	2.88, 5.15	< 0.001
6	4.74	3.58, 6.38	< 0.001
**Gender**			
Male	Reference		
Female	1.54	1.35, 1.75	<0.001
**School location**			
Urban	1.57	1.38, 1.79	<0.001
Rural	Reference		

OR, Odds Ratio; CI, Confidence Interval.

## 4 Discussion

Vision impairment is a prominent public health concern worldwide. It can hinder adults’ work participation and productivity, elevate the risks of depression and anxiety, so that most individuals surveyed believe that good vision is vital to overall health (3, 19, 20). Vision impairment is also a risk factor for subjective cognitive decline and fall-related injuries among the elderly, thereby increasing the demand for care services (21–23). Furthermore, it can adversely affect the academic performance of school-age children; in severe cases, it may lead to delays in language, motor, social, emotional, and cognitive development, ultimately impacting the child’s self-esteem (24, 25). Studies have indicated that even mild vision impairment, if not identified promptly, can significantly diminish an individual’s quality of life (26). Uncorrected refractive error, particularly myopia, a primary cause of vision impairment in children, is both preventable and treatable (27). In large-scale school-based screenings where cycloplegic refraction is often not feasible, reduced VA (defined as UCVA < 20/30 in either eye) has been shown to be a reliable indicator for identifying children at high risk of myopia, demonstrating satisfactory sensitivity and specificity (28). The prevalence of reduced VA is influenced by various factors, including age, sex, geographic region, and socioeconomic status (29). While data on childhood vision health from urban and economically developed regions in China are increasingly available (14, 16, 19), evidence from remote, high-altitude, and less developed areas in western China remains scarce. Therefore, this study aimed to determine the prevalence of reduced UCVA among school-aged children in Wuqia County, a high-altitude region in western China.

High altitude significantly affects both ocular structure and biological function (30). Located in the far western China, Wuqia County has an average altitude of 3,197 meters, characterized by an arid climate and intense ultraviolet radiation. The county has a relatively underdeveloped economy, with a nominal GDP of 5.75 billion yuan in 2024. It covers an area of approximately 22,000 km^2^ and has a resident population of about 62,600. The population is predominantly composed of the Kyrgyz ethnic group, accounting for over 70% of the total. However, the influence of these distinctive geographical and demographic attributes on vision remains unexplored. Our study found that the overall prevalence of reduced VA among school-aged children in Wuqia County was 21.52%. This prevalence is higher than the 18.6% prevalence of UCVA impairment reported among upper-middle socioeconomic status school-aged children in Kathmandu, Nepal, in 2008 (31), but it is significantly lower than the rates reported in other urban areas in China. For example, the prevalence of reduced UCVA among children aged 6–15 years in Changsha, was as high as 46.5% in 2018 (16), while the prevalence of poor vision among school-aged children in Wuhan exceeded 45.7% in 2018–2021 (14). These findings are consistent with research by Jan et al., which reported a higher prevalence of vision impairment in more economically developed regions of China compared to less affluent areas (32). This disparity may be attributable not only to economic factors but also to the unique geographical environment of Wuqia County.

Our results indicate a higher prevalence of reduced VA in female students (26.62%) compared to their male peers (16.90%). And this disparity widened with increasing grade level. This observation aligns with the sex-based differences reported by Ma et al. in a study of Chinese minority students (33). Similarly, numerous other studies across different regions and populations have reported consistent findings (11, 13, 34). Several factors may contribute to this disparity, including genetic predisposition, environmental influences, and lifestyle habits - such as longer time spent reading and less outdoor activity among females. Future studies should investigate these potential contributors to inform the development of tailored preventive strategies.

We observed a notable trend of increasing prevalence of reduced VA with grade level, from 8.10% in grade 1–31.03% in grade 9. This finding is consistent with previous studies. For instance, the prevalence of myopic visual impairment increased from 10.9% in 10-year-olds to 27.3% in 15-year-olds in Kathmandu (31). Similarly, a study in Bhutan demonstrated a progressive decline in normal visual acuity (UCVA ≥ 20/30) with advancing school grade, from 85.6% in grade IV to 78.5% in grade IX (35). This escalating prevalence underscores the importance of early intervention, as timely vision screening enables prompt corrective measures. Such measures can mitigate the severity of vision impairment and its potential long-term adverse effects on academic performance and overall quality of life. Recognizing this critical issue, the Chinese government has prioritized it and implemented relevant measures.

Another key finding of our study is the difference in the prevalence of reduced VA between urban and rural primary schools. The prevalence was significantly higher in urban primary schools (21.33%) than in rural schools (15.32%). This trend is similar to the previous findings reported by Srivastava et al. in northern India, who observed a comparable pattern (22.14% vs. 12.71%) following the COVID-19 pandemic (36). The higher prevalence of reduced VA in urban areas may be attributed to factors such as greater exposure to digital screens, less time spent outdoors, and more time dedicated to homework - all recognized risk factors for vision impairment. In contrast, rural environments likely offer more opportunities for outdoor activities, which may help mitigate these risks. Future research in this region should directly assess these behavioral and environmental factors to confirm their role in the observed disparity.

Multivariate logistic regression analysis further substantiated the univariate findings, identifying higher grade level, female gender, and urban school location as independent risk factors for reduced VA. The graded increase in risk with advancing school grade, evidenced by the rising odds ratios, underscores the profound impact of prolonged engagement in near-work activities and academic demands associated with higher grades. The significantly elevated risk observed in female students aligns with previous studies and may be influenced by a combination of behavioral, hormonal, and genetic factors that warrant further investigation. Similarly, the independent association of urban residence with reduced VA highlights the potential role of lifestyle differences, such as less time spent outdoors and greater exposure to digital screens in urban settings, even after adjusting for grade and gender. These findings collectively emphasize the multi-factorial nature of reduced VA and the need for interventions that are tailored not only to grade level but also to specific gender and geographical contexts.

This study has several limitations inherent to its cross-sectional design. Firstly, due to a vast number of individuals being screened, our study does not use cycloplegia. Previous research indicates that non-cycloplegic refraction can overestimate the prevalence of vision impairment in children (37). Secondly, due to constraints of time and funding, refractive error testing was not performed for children with reduced VA to determine the specific underlying causes, which would have enabled more targeted treatment. Thirdly, our analysis did not account for other potential influencing factors, such as ethnicity, which may affect students’ vision. Lastly, data on the severity of reduced VA were not available.

## 5 Conclusion

In conclusion, the present study provides the first large-scale vision screening data for school-age children residing in the high altitude regions of western China. It reveals a significant burden of reduced VA, with higher grade level, female gender, and urban school location identified as independent risk factors. Given the notable prevalence of reduced VA in this population, it is imperative for the Chinese government to implement targeted intervention strategies. Vision screening programs should be strengthened through the expansion of their scope and frequency. It is recommended that screenings commence early and continue with annual assessments throughout the school years to monitor visual development and facilitate timely intervention. Future research should focus on elucidating the specific etiology and modifiable risk factors for reduced VA in this high altitude setting. Key investigative priorities should include quantifying detailed digital device use, homework duration, and time spent outdoors among children in Wuqia County. A deeper understanding of these behavioral and environmental determinants is crucial for developing targeted, evidence-based, and culturally appropriate preventive strategies.

## Data Availability

The original contributions presented in this study are included in this article/supplementary material, further inquiries can be directed to the corresponding author.

## References

[1] Brown R Barrett A. Visual impairment and quality of life among older adults: an examination of explanations for the relationship. *J Gerontol B Psychol Sci Soc Sci.* (2011) 66:364–73. 10.1093/geronb/gbr015 21402645

[2] Frick K Joy S Wilson D Naidoo K Holden B. The global burden of potential productivity loss from uncorrected presbyopia. *Ophthalmology.* (2015) 122:1706–10. 10.1016/j.ophtha.2015.04.014 26190438

[3] Naidoo K Fricke T Frick K Jong M Naduvilath T Resnikoff S Potential lost productivity resulting from the Global burden of Myopia: systematic review, meta-analysis, and modeling. *Ophthalmology.* (2019) 126:338–46. 10.1016/j.ophtha.2018.10.029 30342076

[4] Burton M Ramke J Marques A Bourne R Congdon N Jones I The lancet global health commission on global eye health: vision beyond 2020. *Lancet Glob Health.* (2021) 9:e489–551. 10.1016/S2214-109X(20)30488-5 33607016 PMC7966694

[5] GBD 2019 Blindness and Vision Impairment Collaborators, Vision Loss Expert Group of the Global Burden of Disease Study. Trends in prevalence of blindness and distance and near vision impairment over 30 years: an analysis for the Global burden of disease study. *Lancet Glob Health.* (2021) 9:e130–43. 10.1016/S2214-109X(20)30425-3 33275950 PMC7820390

[6] Pascolini D Mariotti S. Global estimates of visual impairment: 2010. *Br J Ophthalmol.* (2012) 96:614–8. 10.1136/bjophthalmol-2011-300539 22133988

[7] Bruce A Fairley L Chambers B Wright J Sheldon T. Impact of visual acuity on developing literacy at age 4-5 years: a cohort-nested cross-sectional study. *BMJ Open.* (2016) 6:e010434. 10.1136/bmjopen-2015-010434 26883240 PMC4762077

[8] Brown M Brown G Sharma S Busbee B. Quality of life associated with visual loss: a time tradeoff utility analysis comparison with medical health states. *Ophthalmology.* (2003) 110:1076–81. 10.1016/S0161-6420(03)00254-9 12799229

[9] Morgan I Ohno-Matsui K Saw S. Myopia. *Lancet.* (2012) 379:1739–48. 10.1016/S0140-6736(12)60272-4 22559900

[10] Sheeladevi S Seelam B Nukella P Borah R Ali R Keay L. Prevalence of refractive errors, uncorrected refractive error, and presbyopia in adults in India: a systematic review. *Indian J Ophthalmol.* (2019) 67:583–92. 10.4103/ijo.IJO_1235_18 31007213 PMC6498913

[11] Prakash W Marmamula S Mettla A Keeffe J Khanna R. Variations in the prevalence of vision impairment across regions among school children in Telangana State. *South India. Indian J Ophthalmol.* (2023) 71:3322–7. 10.4103/IJO.IJO_215_23 37787229 PMC10683682

[12] Duelund N Nisted I Frederiksen I Eika Jørgensen M Heegaard S Jensen H. Vision screening of school children in greenland 2017-2022: coverage and low vision prevalence. *Int J Circumpolar Health.* (2024) 83:2403221. 10.1080/22423982.2024.2403221 39283053 PMC11407415

[13] Chen W Fu J Sun A Li L Sun Y Meng Z. Paediatric vision screening in Urban Lhasa from the Tibetan Plateau of Southwest China. *Eye.* (2023) 37:1336–41. 10.1038/s41433-022-02126-y 35668139 PMC10170070

[14] Wang W Peng S Zhang F Zhu B Zhang L Tan X. Progression of vision in chinese school-aged children before and after COVID-19. *Int J Public Health.* (2022) 67:1605028. 10.3389/ijph.2022.1605028 36032274 PMC9402781

[15] Thorisdottir R Faxén T Blohmé J Sheikh R Malmsjö M. The impact of vision screening in preschool children on visual function in the Swedish adult population. *Acta Ophthalmol.* (2019) 97:793–7. 10.1111/aos.14147 31127702

[16] Xiang S Zhao S Li X Li L Xie L Kang R The prevalence of reduced visual acuity in children from an urban district in China from 2002 to 2018. *Eye.* (2021) 35:2550–5. 10.1038/s41433-020-01269-0 33188291 PMC8376935

[17] Donahue S Baker C Committee on Practice and Ambulatory Medicine, American Academy of Pediatrics, Section on Ophthalmology, American Academy of Pediatrics, American Association of Certified Orthoptists, American Association for Pediatric Ophthalmology and Strabismus, Procedures for the evaluation of the visual system by pediatricians. *Pediatrics.* (2016) 137:1–9. 10.1542/peds.2015-3597 26644488

[18] Furtado J Lansingh V Carter M Milanese M Peña B Ghersi H Causes of blindness and visual impairment in Latin America. *Surv Ophthalmol.* (2012) 57:149–77. 10.1016/j.survophthal.2011.07.002 22137039

[19] Zhao X Liu W Lu B Zhu X Zhou M Sun X. Visual impairment and depression in China: a 7-year follow-up study from national longitudinal surveys. *BMJ Open.* (2022) 12:e055563. 10.1136/bmjopen-2021-055563 35477885 PMC9047878

[20] Scott A Bressler N Ffolkes S Wittenborn J Jorkasky J. Public attitudes about eye and vision health. *JAMA Ophthalmol.* (2016) 134:1111–8. 10.1001/jamaophthalmol.2016.2627 27490785

[21] Luo L Jiang N Zheng X Wang P Bi J Xu F Effect of visual impairment on subjective cognitive decline in older adults: a cross-sectional study in China. *BMJ Open.* (2024) 14:e072626. 10.1136/bmjopen-2023-072626 38688669 PMC11086556

[22] Alsaleh H AlObaidi S Alsaber A. Severity of fall-related injuries and older persons’ hospital admission in Kuwait: a cross-sectional study. *J Frailty Aging.* (2024) 13:565–71. 10.14283/jfa.2024.76 39574283

[23] Larsen P Thiele S Krohne T Ziemssen F Krummenauer F Holz F Visual impairment and blindness in institutionalized elderly in Germany. *Graefes Arch Clin Exp Ophthalmol.* (2019) 257:363–70. 10.1007/s00417-018-4196-1 30483949

[24] Toledo C Paiva A Camilo G Maior M Leite I Guerra M. Early detection of visual impairment and its relation to academic performance. *Rev Assoc Med Bras.* (2010) 56:415–9. 10.1590/s0104-42302010000400013 20835637

[25] Zhou Z Zhu Y Luo R Chen K Li X Guo X The associations of self-perception, movement competence, and clinical features of young school-aged children with glaucoma. *Eur J Pediatr.* (2024) 183:885–95. 10.1007/s00431-023-05262-z 37864600

[26] Cumberland P Rahi J UK Biobank Eye and Vision Consortium. Visual function, social position, and health and life chances: the UK Biobank study. *JAMA Ophthalmol.* (2016) 134:959–66. 10.1001/jamaophthalmol.2016.1778 27466983

[27] Ambrosino C Collins M. Challenges and opportunities of vision screening and refractive error management for underserved children in the United States. *J Binocul Vis Ocul Motil.* (2024) 74:113–7. 10.1080/2576117X.2024.2348266 39882637

[28] Sun H Li A Xu Y Pan C. Secular trends of reduced visual acuity from 1985 to 2010 and disease burden projection for 2020 and 2030 among primary and secondary school students in China. *JAMA Ophthalmol.* (2015) 133:262–8. 10.1001/jamaophthalmol.2014.4899 25429523

[29] Liang J Pu Y Chen J Liu M Ouyang B Jin Z Global prevalence, trend and projection of myopia in children and adolescents from 1990 to 2050: a comprehensive systematic review and meta-analysis. *Br J Ophthalmol.* (2025) 109:362–71. 10.1136/bjo-2024-325427 39317432

[30] Wang Y Yu X Liu Z Lv Z Xia H Wang Y Influence of hypobaric hypoxic conditions on ocular structure and biological function at high attitudes: a narrative review. *Front Neurosci.* (2023) 17:1149664. 10.3389/fnins.2023.1149664 37229428 PMC10203194

[31] Sapkota Y Adhikari B Pokharel G Poudyal B Ellwein L. The prevalence of visual impairment in school children of upper-middle socioeconomic status in Kathmandu. *Ophthalmic Epidemiol.* (2008) 15:17–23. 10.1080/09286580701772011 18300085 PMC6031131

[32] Jan C Xu R Luo D Xiong X Song Y Ma J Association of visual impairment with economic development among chinese schoolchildren. *JAMA Pediatr.* (2019) 173:e190914. 10.1001/jamapediatrics.2019.0914 31058915 PMC6503578

[33] Ma R Bicheng J Zhiqiang J. Analysis of the visual acuity characteristics of Chinese minority primary and middle school students (Article in chinese). *Chin J Sch Health.* (2019) 40:426–9. 10.16835/j.cnki.1000-9817.2019.03.030

[34] Wang J Ying G Fu X Zhang R Meng J Gu F Prevalence of myopia and vision impairment in school students in Eastern China. *BMC Ophthalmol.* (2020) 20:2. 10.1186/s12886-019-1281-0 31898504 PMC6941318

[35] Sharma I Lepcha N Lhamo T Ellwein L Pokharel G Das T Visual impairment and refractive error in school children in Bhutan: the findings from the Bhutan school sight survey (BSSS 2019). *PLoS One.* (2020) 15:e0239117. 10.1371/journal.pone.0239117 32925975 PMC7489552

[36] Srivastava T Kumar A Shukla E Singh V Anuranjani L. Prevalence of refractive errors among school-going children in urban versus rural areas. *Cureus.* (2024) 16:e59197. 10.7759/cureus.59197 38807816 PMC11131348

[37] Chen J Xie A Hou L Su Y Lu F Thorn F. Cycloplegic and noncycloplegic refractions of Chinese neonatal infants. *Invest Ophthalmol Vis Sci.* (2011) 52:2456–61. 10.1167/iovs.10-5441 21087952

